# Predicting Durable Anti-tumor Immune Responses after Cancer Vaccines

**DOI:** 10.33696/cancerimmunol.8.119

**Published:** 2026

**Authors:** John B. Liao, Mary L. Disis

**Affiliations:** 1 Division of Gynecologic Oncology, Department of Obstetrics and Gynecology, University of Washington, 1959 NE Pacific St, Seattle, WA 98195, USA; 2 Cancer Vaccine Institute, University of Washington, 850 Republican St, Seattle, WA 98109, USA

## Immunologic Memory for Cancer Vaccine Efficacy

Recent reports and ongoing clinical trials demonstrate potential roles for cancer vaccine therapy in a number of malignancies [1]. Adaptive cellular immune responses are required for protective immunity when vaccines are used against infectious diseases [2]. For therapeutic cancer vaccines used to treat established tumors, elaboration of durable T cell mediated responses are also understood to be necessary for efficacy [3–5]. Immune responses measured in peripheral blood lymphocytes correlate with clinical outcomes [6,7]. Persistent immunity in the form of memory T cells specific to cancer antigens has been shown to lead to effective antitumor immunity [8]. Antigen-specific T-cell responses after vaccination has correlated with survival in multiple cancers [9–14].

The importance of antigen specific memory T cells for cancer vaccine efficacy highlights the need for improved understanding of biomarkers to optimize vaccine therapies for cancer. Detecting pre-existing immunity to antigens included in a cancer vaccine may select candidates for vaccination. The timing and magnitude of an antigen-specific immune response to vaccination may identify patients who will develop productive and persistent anti-tumor immunity.

## Pre-existing Immunity to Vaccine Antigens

Developing effective antigen specific T cells after vaccination depends on antigen-specific T-cell precursor frequencies [15,16]. Pre-existing CD8 T-cell memory will influence the magnitude of naïve CD8 T cell responses [17]. Acquisition of an effector memory cell phenotype will depend on the CD8 T-cell differentiation state, which is also determined by preexisting memory T cells [18]. Levels of preexisting antigen specific T cells could conceivably be either a positive or negative predictor for persistent antitumor immunity. Higher precursor frequency could lead to more rapid clonal expansion of effector and memory T cells when vaccines are introduced, accelerating antitumor responses. More precursors may not always provide an advantage as there may be a threshold beyond which there may be decreased responses due to intraclonal competition, although this may also be overcome with CD4 T cell help [19,20]. Conversely, high numbers of antigen specific T cells in the presence of known tumor burden or disease progression could represent the inability of an antigen to serve as an immunogenic target, possibly due to immune evasion through the loss of the target on tumor cells.

In our study of vaccines targeting HER2, we found that patients with higher pre-existent T-cell responses to HER2 were more likely to develop higher HER2 T-cell responses. We also found that these antigen specific T cell responses were more likely to last greater than 6 months after immunization in patients with pre-existent immunity than those that did not. Sixty-three percent of patients with baseline antigen specific T immunity against HER2 continued to have responses 1 year after completing HER2 targeted vaccines and the presence of preexisting immunity was associated with a 0.33 log increase [21].

Preexisting immunity to the vaccine vector, rather than the target antigen may be a particular consideration for some cancer vaccines. This may be particularly true for vaccines utilizing bacterial or viral vectors, as the subsequent immune response may be modified due to more rapid clearance of the vaccine [22]. Preexisting immunity to viral vectors such as adenovirus have been an obstacle to generating immune responses because they can reduce the time and exposure to the vaccinated antigens, but this has also been overcome to generate antitumor immunity [23,24].

## Timing and Magnitude of Response to Vaccination

We have found that the magnitude of antigen-specific T cell elaborated one month after a vaccine series is more predictive of long term immunity than the existence of pre-existing immune responses [21]. Patients with HER2+ cancers were treated with HER2 directed vaccines and antigen specific T-cells were measured by interferon-gamma ELISPOT assays at baseline, one month after completing vaccine series, and long-term follow-up at 1 year. One month after vaccination, an increase in HER2+ interferon-gamma T-cell frequency was associated with retaining those immune responses long term. A one-log increase of month-1 T-cell response was associated with 0.47-log increase in long term T-cell responses. Patients who had immunity one month after completing the vaccination series had higher odds of having persistent HER2-specific immunity, OR=6.50 (95%CI 2.41-17.52, p=0.0002), regardless of whether they had pre-existing immunity.

Signaling through interferon-gamma, can control the magnitude of T-cell responses and memory differentiation [25]. However, although interferon-gamma is produced by cytotoxic T cells and is associated with a desired Th1 response for antitumor immunity, it is also associated with synthesis of immune checkpoint molecules and immunosuppressive mechanisms [26]. Therefore the magnitude and timing, in addition to the phenotype of the elaborated immune response to vaccine are all important considerations for long-lasting immunity.

Depending on the vaccine, T cell frequencies are generally expected to peak between days 7 to 14 before contracting with a very small percentage of T cells persisting as memory T cells [27]. Therefore, the 1 month timepoint may assess the earliest measures of initial clonal expansion that establishes a memory pool ofT cells, capturing the transition from effector to early memory T cells representing a post contraction population that is potentially long-lived [28]. The magnitude of this response is related to the frequency of antigen specific T cells in the preexisting repertoire, how many are activated and start to divide with vaccination and how many cells survive the contraction phase. With each successive vaccination in a series, the precursor frequency would be expected to increase along with the subsequent population of memory T cells, which should represent antitumor T cell immunity. Meta-analysis of TCR engineered T cell trials suggest that clinical responses will correlate with higher numbers of antigen specific effector T cells [29]. Mathematical modeling taking into account T cell priming, migration, and interaction with tumors based on these data support the existence of a threshold of effector T cell needed to control tumor progression [29].

A third of patients in our study did not develop antigen specific immunity one month after vaccination [21]. For patients that do not develop immunity after vaccination, a number of factors may be at play. These may include the current immune microenvironment of the tumor, the immune phenotype of the T cells available for a vaccine, and the suitability of a patient’s tumor and immune system for a particular vaccine. The genotype and immune repertoire of patient’s individual immune system may favor a response to vaccines on the basis of HLA restriction of the vaccine epitopes or the presence of pre-existing immunity to the vectors or adjuvants.

## Conclusions

**Figure 1.**
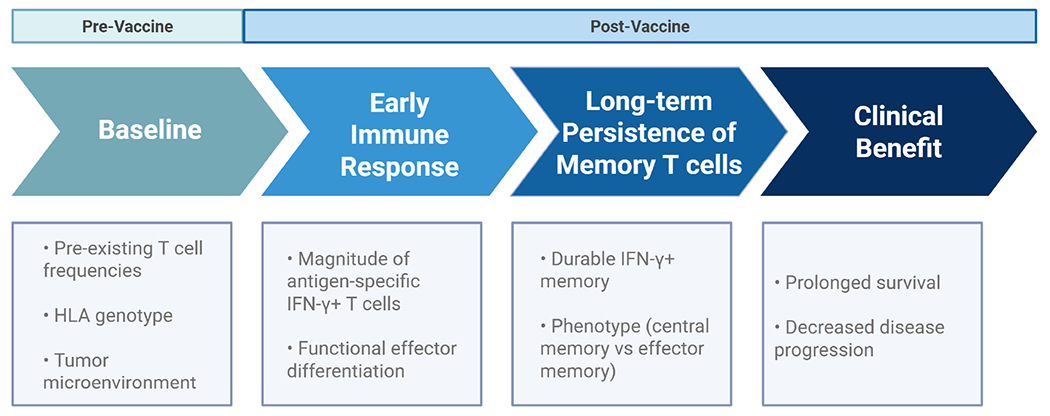
Proposed predictive timeline linking pre-vaccination determinants to clinical benefit following therapeutic vaccination.

Predictive markers for long term immunity from cancer vaccines require further study to optimally deploy vaccines. The journey from vaccination to generation of effector T cells capable of trafficking to tumors and overcoming a hostile tumor microenvironment may also be influenced by the immune properties of the specific cancer, the patient’s own immune repertoire, in addition to factors we have discussed. Increasing our understanding of these immune dynamics, and how they can be modulated will allow the development of rational strategies to incorporate cancer vaccines into cancer treatment paradigms. As the development of cancer vaccines accelerate, markers that predict long-lasting anti-tumor immunity become an ever more important consideration. Factors that span the timeline from before a patient receives a vaccine to clinical responses (Figure 1) are ripe for further study and could be explored further as a composite prediction model integrating pre-existing factors with early immune responses, and tumor microenvironment biomarkers. Identifying and monitoring the key immune predictors of effective antitumor immunity could permit optimal integration of vaccines with adjuvants, boosters, and other immunotherapies such as immune checkpoint blockers which has been noted in several clinical trials [7,30–32]. These agents could further synergize with vaccines and help overcome barriers to efficacy such as immunosuppressive tumor microenvironments and factors that influence T cell exhaustion, expansion and persistence.
